# Therapeutic Benefit of *Vernonia amygdalina* in the Treatment of Diabetes and Its Associated Complications in Preclinical Studies

**DOI:** 10.1155/2023/3159352

**Published:** 2023-11-22

**Authors:** Du-Bois Asante, Gideon Akuamoah Wiafe

**Affiliations:** ^1^Department of Biomedical Sciences, University of Cape Coast, Ghana; ^2^Department of Forensic Science, University of Cape Coast, Ghana

## Abstract

Diabetes mellitus (DM), a complex heterogeneous metabolic disorder characterized by a defect in the function of insulin, is on the rapid rise globally. Sustained hyperglycemia which is a major sign of DM is linked to the generation of reactive oxygen species which promotes adverse complications of the disorder. Traditional herbal treatment of DM is a common practice in Africa and other tropical parts of the world. *Vernonia amygdalina* (VA), one of the highly researched species in the Asteraceae family, has proven to possess potent antidiabetic properties. Several phytochemicals identified in multiple extracts from VA are purported to be responsible for the antidiabetic potential of the plant. In this review, we discuss the therapeutic potential of VA in diabetes and its associated complications. We appraise the current evidence and further suggest potential areas that could be effectively exploited in future VA research on diabetes.

## 1. Introduction

Diabetes mellitus (DM) is a chronic heterogeneous metabolic disorder represented by the incidence of hyperglycemia due to a defect in function or secretion of insulin [1]. There are two major DM types: type 1 and type 2 diabetes. Type 1 DM is due to an autoimmune disease that leads to the destruction of insulin-producing pancreatic *β*-cells. This usually results in low or no insulin, causing hyperglycemia. On the other hand, type 2 DM, the more common type, which is more prevalent [2], is defined by the inability of the body to use the insulin secreted as a result of insulin resistance [3].

According to the World Health Organization (WHO), about half a billion individuals are living with the disorder globally. The majority of these individuals are found in low- and middle-income countries [4]. Though there is a steady increase in the prevalence globally, low- and middle-income countries seem to have a rapid increase and it is projected that there will be a 21.1% increase by 2045 [5]. In sub-Saharan Africa, increased modernization has contributed to the sharp surge in recorded diabetes cases [6]. Several chronic complications result from untreated and improper treatment of diabetes. These complications range from acute to chronic forms. The acute form of DM complications includes diabetic ketoacidosis (DKA) and nonketotic hyperosmolar state (NKHS), while the chronic form includes several microvascular (nephropathy, neuropathy, and retinopathy), macrovascular (cerebrovascular disease, coronary artery disease, and peripheral vascular disease), and nonvascular (sexual dysfunction, skin complications) [7, 8] forms and, if left unattended, are likely to lead to death [3, 9].

Dietary and lifestyle modifications play a huge role in the treatment of type 2 DM [3, 10]. However, due to the severity of DM especially at the late stage, several treatment options have been developed to curtail the complications. These treatment options are not without side effects and may lead to further dilapidating problems such as severe hypoglycemia [9].

Many herbal plants have antidiabetic properties, and their use especially in developing countries is on the rise [11]. Bioactive compounds from natural plant products such as flavonoids, tannins, terpenoids, saponins, carotenoids, alkaloids, terpenes, chromane, and glycosides have been shown to possess several antidiabetic properties [12–15]. A review by Odeyemi and Bradley revealed Asteraceae as the prominent family of plants used to treat diabetes in South Africa. One highly researched species from the Asteraceae family is *Vernonia amygdalina* (VA) [16]. Here, we explored the literature in NCBI, PubMed, Embase, and MEDLINE and summarized the therapeutic benefits of VA in the treatment of diabetes and its associated complications. We further discussed emerging concepts in research in the field of herbal medicine that could potentially galvanize the utilization of VA in the near future, for the effective treatment of diabetes and its related complications.

## 2. Description of Plant and Its Traditional Use

**Figure 1:**
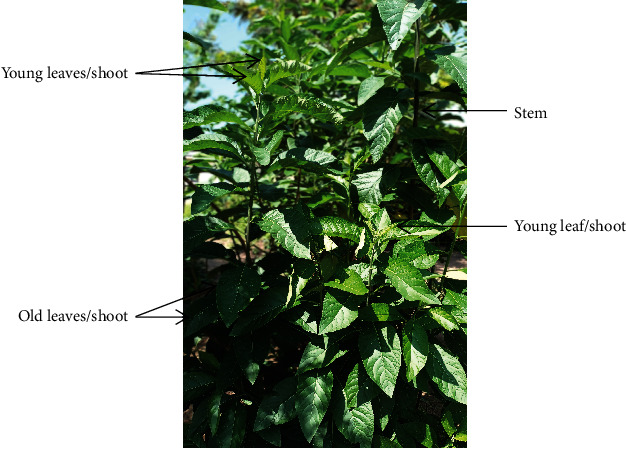
*Vernonia amygdalina* plant showing leaves and stem.

*Vernonia amygdalina* (VA) is commonly known as bitter leaf due to its bitter taste. It is a small shrub that grows between 3 and 7 meters high and mostly found in tropical Africa and also grown in other Asian countries [17–19]. Other names of the plant and its classification are presented in Tables 1 and 2, respectively. The leaves are medium to dark green, having an apex and a tapering base. It is usually 10–15 × 4–5 cm long. It has an entire margin and is lanceolate to oblong in shape [20]. Traditionally, the leaves of VA (Figure 1) are commonly used as an appetite stimulant and as a vegetable in stews and soups. It is normally washed with clean water to reduce the bitterness before use in food preparation in West Africa [21].

Extracts from the leaves and roots are also used in the indigenous treatment and management of several diseases such as malaria and diabetes [22, 23], as well as anticancer [24], antimicrobial [25], antileishmanial [26], antifertility [27], anti-inflammatory, antipyretic [28], analgesic [29], appetizer [30], laxative [31], oxytocin [32], and wound healing [33]. Among all these reported traditional usages, its usage as antidiabetic agent is widely acclaimed [22, 34, 35]. This ethnopharmacological ability of VA in the treatment of diabetes has been evaluated and confirmed in several experimental models using different and similar extraction solvents (Table 3).

Since VA is easily accessible and used in various food preparations, it can be an economical source of medication for the treatment of diabetes [41]. Nevertheless, extract dosage which could prove to be fatal when used excessively is the main factor hindering the wide usage of these medicinal plants in the local communities.

## 3. Therapeutic Effects of Vernonia amygdalina: Experimental Models

### 3.1. Hypoglycemic Effect

**Figure 2:**
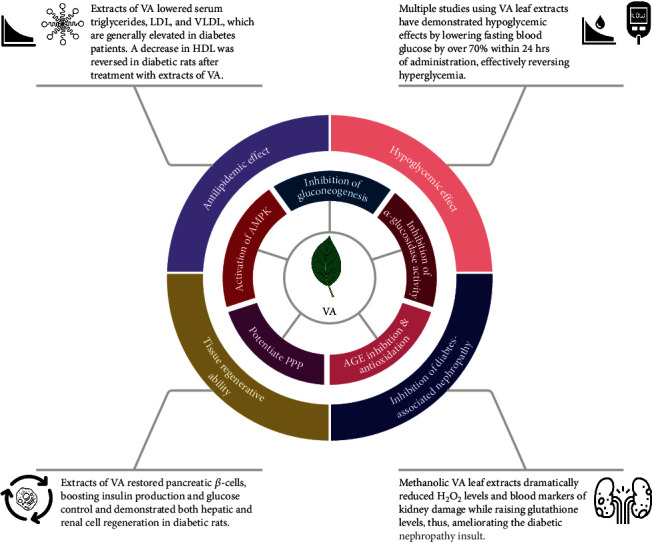
Summary of the therapeutic potential of *Vernonia amygdalina* (VA) in diabetes and its associated complications.

Decoction of the leaves of VA has been used for indigenous treatment of diabetes [55]. Several studies involving experimental models (Table 3) have confirmed the marked hypoglycemic effect of the extracts of VA (Figure 2). In a recent study [40], an 80% methanol extract of VA was fractionated in an increasing polarity of solvents (n-hexane, chloroform, ethyl acetate, n-butanol, and water). A total of 9 components (C1-C9) obtained from the chloroform fraction were tested for their antidiabetic property on Wistar Albino rats weighing 150-200 g. Animals with nonfasting blood glucose greater than 300 mg/dl after induction of diabetes with diet-fed streptozotocin-treated were considered diabetic (type 2 diabetes) and incorporated into the study. The components were purified and administered by oral intubation at a dose of 10 mg/kg b.w. After treatment of the diabetic rats with the components, component 5 (C5) was found to exhibit the highest hypoglycemic effect of 12.55% as compared to metformin-treated diabetic rats (18.07%). A spectroscopic analysis of C5 revealed that the compound is 11*β*,13-dihydrovernolide [40]. When analysing 12 common leafy culinary vegetables (*Amaranthus hybridus*, *Gnetum africanum*, *Gongronema latifolium*, *Ocimum gratissimum*, *Pterocarpus mildbraedii*, *Pterocarpus santalinoides*, *Piper guineense*, *Solanum macrocarpon*, *Talinum triangulare*, *Telfairia occidentalis*, *Vernonia amygdalina*, and *Vitex doniana*) consumed in Nsukka, Southeastern Nigeria, for their hypoglycemic potential, VA showed significantly high antidiabetic activity, compared with the other plants. VA significantly lowered fasting blood glucose level by 70.6% (520.00 ± 7.80 mg/dl-150.00 ± 2.16 mg/dl) 24-hour posttreatment [44]. Other studies [34, 43–47, 52, 54, 56] have also reported similar hypoglycemic effects of VA using experimental models in different regions globally. All these studies consistently demonstrate the potent hypoglycemic potential of VA, depicting that it could be exploited further for its clinical utilization in diabetic cases.

### 3.2. Antilipidemic Effect in Diabetes-Associated Dyslipidemia

Diabetic dyslipidemia refers to the disruption in the levels of serum lipids, i.e., low high-density lipoprotein cholesterol (HDL-C), elevated low-density lipoprotein cholesterol (LDL-C), increased VLDL-triglycerides, and the prevalence of small dense LDL [57, 58]. This is a major risk factor for cardiovascular diseases [57]. Nwanjo [53] investigated the hypolipidemic effects of aqueous extracts of VA in streptozotocin-induced diabetic adult Wistar Albino rats. The diabetic rats were treated with 200 mg/kg aqueous extract of VA twice a day for 14 days. Upon analysis, serum triglycerides and LDL were significantly lower in the normal and treated rats as compared to diabetic control rats. Another study by Asante et al. [13] revealed the hypolipidemic potential of VA. STZ-induced diabetic rats were treated with ethanolic extracts of young and old leaves of VA in dose ranges of 10, 30, and 300 mg/kg b.w. Major signs of diabetic dyslipidemia were observed in diabetic control rats. However, among the treated rats, there was a significant increase in HDL-C, popularly known as good cholesterol, which has the potential to reduce an individual's risk of cardiovascular diseases [59]. A significant decrease in LDL-C and VLDL-C was also observed [13]. Another study also reported similar results indicating the antilipidemic ability of VA [52]. In yet another study, serum total cholesterol, triacylglycerol, LDL-C, and serum total cholesterol/HDL-C which were significantly high were restored to normal levels after a 28-day treatment with chloroform fraction of VA [46].

All these studies further establish the antilipidemic potential of VA in diabetes-associated dyslipidemia.

### 3.3. Treatment of Diabetes-Associated Nephropathy

Diabetic nephropathy has become the leading cause of end-stage renal failure in both developed and developing countries. About 20%-40% of all diabetic patients usually suffer from diabetic nephropathy, however, if left untreated, 80% and 40% of type 1 and type 2 diabetic individuals, respectively, are likely to develop definite nephropathy [60]. In diabetic patients, it usually arises from chronic hyperglycemia which affects mesangial cells causing pathological changes such as their proliferation, matrix formation, and thickening of the basement membrane [60, 61]. Chronic hyperglycemia increases the production of reactive oxygen species (ROS) and a concurrent weakening of antioxidant defence mechanisms, leading to oxidative stress. Tissue damage resulting from chronic hyperglycemia has been linked to different mechanisms ranging from advanced glycosylation, activation of the protein kinase C pathway, and acceleration of the aldose reductase pathway. Oxidative stress occurs in all these pathways [60]. VA is known to have antioxidant properties and might have the potential to reverse the mechanisms that lead to tissue damage in diabetic nephropathy. Assessing this ability, Adeoye et al. [62] induced diabetes in male Wistar rats (150 g-200 g) with freshly prepared alloxan monohydrate (100 mg/kg) intraperitoneally. After 28 days of treatment, methanolic leaf extracts of VA (MLVA) (200 mg/kg and 400 mg/kg) significantly reduced the levels of serum markers of kidney damage (creatinine and blood urea nitrogen (BUN)) which were found to be in high levels in the diabetic control rats. Okoduwa et al. [46] also reported similar findings using the different fractions of the leaves of VA. Also, high H_2_O_2_ levels and malondialdehyde observed in diabetic control rats showed decreased levels when compared with the MLVA-treated group. The major antioxidant in the kidney, glutathione (GSH), was reduced significantly in diabetic control rats. However, treatment with MLVA restored GSH levels near the normal values indicating the potential antioxidant properties of VA. Activities of glutathione S-transferase (GST) and superoxide dismutase (SOD), major enzymatic antioxidants, were significantly higher in rats treated with MLVA than normal control and diabetic control rats. This study demonstrated that MLVA has the potential to prevent the progression of diabetic nephropathy through its antioxidant effects [62]. Similar observation was reported earlier using VA leaves [63].

### 3.4. Tissue Regenerative Ability of *Vernonia amygdalina*

Chronic hyperglycemia has been associated with increased oxidative stress which plays a significant role in associated complications of diabetes. Damage to hepatic cells as a result of long-term diabetes has been linked not only to oxidative stress but unusual inflammatory responses [64]. The array of pathological defects of diabetic hepatocellular damage includes aberrant activities of liver enzymes, disruption of the hepatic cord, vacuolation of hepatocytes, periportal fibrosis, and bile duct proliferation [65, 66]. VA extracts have proven to ameliorate these pathologic effects in experimental rats. For instance, hepatic lesions such as hepatocellular necrosis, cytoplasmic vacuolation, and infiltration of nonspecific inflammatory cells in diabetic rats were reversed using young and old VA leaves in STZ-induced rats [13]. This was evidenced by increased parameters of the liver enzymes alanine aminotransferase (ALT), aspartate aminotransferase (AST), and alkaline phosphatase (ALP). However, treatment with the doses of the extract showed an array of hepatoprotective functions. The 30 mg/kg of the young leaf extract and 300 mg/kg of both young and old leaf extracts showed pronounced hepatocellular regenerative abilities characterized by normal hepatocellular structure accompanied by very mild leucocytes and oedema. The hepatoprotective and tissue regenerative ability of the extracts may be dose-dependent. Levels of the liver enzymes (ALT, AST, and ALP) showed a significant decrease upon treatment with the extracts of both young and old leaves of VA [13]. Another study by Okoduwa et al. [46] showed that oral administration of leaf fractions of VA has the potential to restore biomarkers of liver function near normal levels after 28-day treatment in diabetic rats.

One significant pathological characterization of DM is pancreatic cell destruction leading to pancreatic *β*-cell destruction in the islets of Langerhans [66, 67]. This causes low or no insulin secretion resulting in high glucose levels [68]. A study by Atangwho et al. [51] revealed the tissue regenerative ability of VA in pancreatic cells in rats. Diabetes was induced by the intraperitoneal injection of 150 mg/kg b.w. of alloxan monohydrate. This resulted in an incredible increase in blood glucose levels indicative of a disruption in insulin secretion and function. Pancreatic tissues from normal control rats showed intact and well-stained islets and acini with no signs of pathological insults. In contrast, tissues from the untreated diabetic rats revealed a complete degeneration of the islets of Langerhans and acinar cells marked by necrosis and fat deposition. On the other hand, tissues from treated diabetic rats showed new zones of well-stained islets and uniform distribution of serous acini cells indicating regeneration. The regeneration complements the reduction in serum glucose levels though not back to normal levels [51]. In yet another study, ethanolic leaf extracts of young and old leaves of VA also showed dose-dependent regeneration of the *β*-cells of the pancreas in STZ-induced diabetic rats. This was evidenced by the reduction in glucose levels posttreatment indicating the potentiation of insulin secretion [13]. In addition, Ong et al. [56] also reported that pancreatic *β*-cells from VA-treated rats showed no vacuolation and less degranulation compared to diabetic control rats. Higher levels of insulin were also detected in this study, and this is indicative of the pancreatic *β*-cell protective effect of VA. Increased insulin sensitivity and similar pancreatic *β*-cell regeneration have also been reported in another study [46]. Thus, these studies show that extracts of VA leaves have pancreatic tissue regenerative ability, and this enhances insulin secretion, hence helping in lowering of blood glucose levels.

Multiple studies [62, 63] have highlighted significant regeneration of the renal tissue microenvironment in diabetic experimental models following VA extract administration. The pathological insults that were significantly reversed include congestion of vessels, hypercellularity and necrosis of glomerulus, degeneration of cells in the glomerular capsule, and widening of the capsular space in the renal corpuscle.

Furthermore, immunohistochemical studies of the renal tissue reported by Adeoye et al. [62] demonstrated significant downregulation of the antiapoptotic marker, Bcl-2, and proinflammatory marker, NF-*κ*B, in the treated rats compared to the negative control group. This demonstrates ameliorative effects induced by the extracts in the renal tissues and, thus, validates the renal regenerative effect reported in the study [62].

Overall, all these studies highlight the potential of VA extract tissue ameliorative effect in diabetic models and hence confirm the restoration of necrotised and degenerated parenchymal cells of the organs in the studies above.

## 4. Molecular Mechanisms of *Vernonia amygdalina* as an Antidiabetic Agent

### 4.1. Inhibition of *α*-Glucosidase Activity

Alpha-glucosidase is an enzyme that catalyzes the hydrolysis of complex carbohydrates to simple sugars like glucose. This leads to increased postprandial blood glucose levels. Due to its activity, it is one of the major enzymes targeted for the treatment of diabetes [69]. Alpha-glucosidase inhibitors play a pivotal role in this regard [9]. The roots and leaf extracts (aqueous, Soxhlet, and ethanol) of VA were investigated for their potential role in the reduction of alpha-glucosidase activity (Figure 2) in vitro [38]. The various extracts, prepared in concentrations of 5, 10, 50, and 100 *μ*g/ml, were incubated with *α*-glucosidase (from *Saccharomyces cerevisiae*) at 0.35 U/ml and 0.1 M PBS (pH 6.8) for 10 min, at 37°C with the addition of 4 mM *p*-nitrophenyl-*α*-D-glucopyranoside (*p*-NPG). After detection of absorbance values at 405 nm for 45 min, the aqueous and Soxhlet root extracts expressed the maximum inhibition potential with an IC_50_ of 5.6 *μ*g/ml and 39.8 *μ*g/ml, respectively. At 5 *μ*g/ml of the aqueous root extract, the enzymatic activity reduced by 48% and a 100% at 100 *μ*g/ml showing an absolute inhibition potential. The aqueous leaf extract also showed inhibition potential in a concentration-dependent fashion [38]. In a more recent study [36], the bioactive compound, luteolin, from VA substantially suppressed *α*-glucosidase activity in a concentration-dependent manner (5, 10, 25, 40, and 50 *μ*M) [36]. Similarly, infusion of VA leaves at concentrations of 15, 30, 60, 120, and 240 *μ*g/ml significantly inhibited *α*-glucosidase activity in a dose-dependent manner indicating the antidiabetic property of VA [41]. Results from these studies consistently show that both root and leaf extracts of VA have high anti alpha-glucosidase activity, confirming their antidiabetic potential.

### 4.2. Advanced Glycation End Product Inhibition and Antioxidation

**Figure 3:**
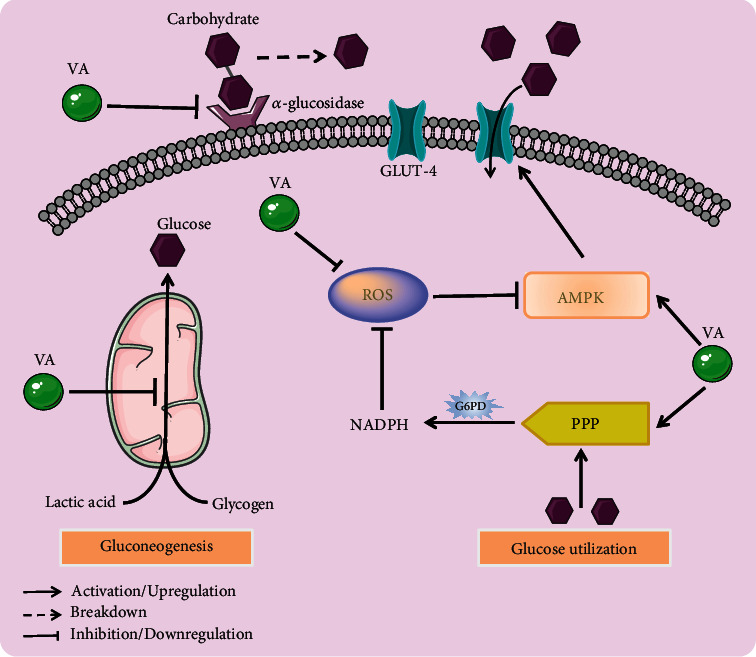
Molecular pathways for the antidiabetic activity of *Vernonia amygdalina* (VA). This figure highlights the various mechanistic pathways by which VA exerts its antidiabetic effect.

The formation of advanced glycation end products (AGEs) in associated complications of diabetes is linked with hyperglycemia and oxidative stress [70]. Reports elsewhere indicate that the accumulation of AGEs leads to the formation of reactive oxygen species (ROS) resulting in glycation stress conditions in diabetes [71, 72]. Thus, inhibition of AGE formation by natural products from daily meals is preferred in the prevention of complications of lifestyle-related diseases like diabetes [73]. In view of this, a study reported remarkable results from the inhibition of ribose-induced BSA glycation by aqueous, Soxhlet, and ethanolic root and leaf extracts of VA after 5 days of observed glycation [38]. This occurred in a concentration-dependent manner (5, 10, 50, 100, and 500 *μ*g/ml). All doses of the extracts showed significant antiglycation effects and proved to be antihyperglycemic agents [38]. Moreover, luteolin (10 *μ*M–100 *μ*M) inhibited AGE formation in ribose-, fructose-, and glucose-induced BSA glycation. This occurred in a concentration-dependent manner with IC_50_ values of 58 *μ*M (−Log IC_50_ = 4.24 ± 0.02), 19.6 *μ*M (−Log IC_50_ = 4.71 ± 0.04), and 40.4 *μ*M (−Log IC_50_ = 4.39 ± 0.05), respectively [36]. In comparison with vernodalol and ascorbic acid, luteolin exhibited a relatively higher antioxidant activity (7.3 ± 1.20 vs. 0.82 ± 0.11 vs. 0.94 ± 0.13 *μ*mol Trolox equivalent antioxidant capacity (TEAC)/*μ*mol compound). Overall, this study showed that the antioxidation and antiglycation potential can be attributed to the presence of the bioactive compound, luteolin [36]. In yet another study, STZ-induced diabetic rats exhibited high levels of malondialdehyde due to increased lipid peroxidation indicating high oxidative stress. Treatment with 200 mg/kg aqueous leaf extract of VA twice a day for 14 days decreased malondialdehyde levels demonstrating its antioxidant properties [53]. Similarly, crude extracts of young and old leaves of VA also exhibited radical scavenging properties (Figure 3) *in vitro* against 2,2-diphenyl-1-picryl hydroxyl (DPPH) radical [13].

### 4.3. Inhibition of Gluconeogenesis

Gluconeogenesis is a series of metabolic reactions that results in the production of new glucose molecules aiding glucose balance in the body, especially in a state of starvation [74, 75]. There are four major enzymes that facilitate these reactions: pyruvate carboxylase, phosphoenol pyruvate carboxykinase (PEPCK), fructose 1,6-bisphosphatase (F16Pase), and glucose 6-phosphatase (G6Pase) [76]. Sustained hyperglycemia observed among diabetic individuals is reported to result from increased gluconeogenesis [77, 78]. Thus, the inhibition or strict regulation of gluconeogenesis in diabetic patients is paramount in the prevention of adverse effects. The expression of gluconeogenic target genes G6Pase, F16BP, and PEPCK was accessed in the liver and muscle tissues of STZ-induced diabetic rats. The rats were treated with metformin (500 mg/kg) and chloroform fraction of VA (200 mg/kg and 400 mg/kg) in half doses twice daily for 14 days. After treatment, the targeted tissues were harvested for gene expression analysis. G6Pase expression in the liver, though not statistically significant, was found in low quantities after the 14-day treatment as compared to the diabetic control group. Induction of diabetes caused a significant increase in the expression of F16BP; however, upon treatment, there was a significant downregulation in the levels of the enzyme. Similar results were observed in the muscle tissues. PEPCK expression was also upregulated upon induction of diabetes in the liver and muscle tissues. Significant reduction in its expression after treatment was observed [48]. The reduced expression of these enzymes is suggestive of gluconeogenesis inhibition by VA. Likewise, when western blot was used to analyse the expression of G6Pase and PEPCK in the liver of STZ-induced Kunming mice (20-25 g), G6Pase and PEPCK proteins significantly increased in the diabetic rats as compared to the control. VA treatment (50, 100, and 150 mg/kg/d) significantly decreased the expression of these proteins [43].

Furthermore, increased tyrosinase and amylase activities have been associated with an increased risk of type 2 DM [79, 80]. These enzymatic activities enhance the generation of glucose. In line with this, Patathananone et al. [37] reported significant antityrosinase and antiamylase activity of VA extracts. Thus, the action of bioactive compounds of VA may be exerted on several targets concurrently and could be key for effective treatment of diabetes and its related complications.

Overall, these studies suggest that inhibition of gluconeogenesis is one of the mechanisms by which VA exerts its antidiabetic potential.

### 4.4. Potentiating the Pentose Phosphate Pathway (PPP)

The expression of glucose-6-phosphate dehydrogenase (G6PD), a major enzyme in the pentose phosphate pathway (PPP), was determined. The PPP through the action of G6PD produces nicotinamide adenine dinucleotide phosphate (NADPH). NADPH plays a role in maintaining cellular antioxidative defence systems by acting as a reducing agent [81, 82]. Hepatic G6PD levels were reduced by 1.79-fold in diabetic rats. Treatment with metformin (500 mg/kg) and chloroform fraction of VA (200 mg/kg and 400 mg/kg) upregulated the levels of the enzyme indicating an appreciable utilization of glucose [48]. These studies suggest that one of the mechanisms by which VA functions as an antidiabetic is by enhancing the PPP activity (Figure 2).

### 4.5. Activation of Adenosine 5′-Monophosphate Kinase (AMPK)

AMPK is a biomolecule that plays a key role in energy homeostasis in the body [83]. It is reported that AMPK inhibits hepatic gluconeogenesis in mitigating diabetes mellitus as it also functions in the regulation of G6Pase and PEPCK [84]. Also, AMPK improves pancreatic *β*-cell survival, decreases insulin resistance, and enhances glucose metabolism and uptake by upregulating the levels of glucose transporter 4 (GLUT-4) [85]. These activities of AMPK further ameliorate the complications of diabetes and are therefore a good target for drug production. The levels of AMPK and phosphorylated AMPK (p-AMPK), i.e., the activated form of AMPK, were measured in STZ-induced (60 mg/kg) diabetic mice after 6 weeks of treatment with VA (50 mg/kg, 100 mg/kg, and 150 mg/kg). Their ratios were calculated, and a significant reduction was observed in the untreated diabetic group (negative control). Treatment with VA increased the phosphorylation of AMPK (Figure 3) as well as the ratio, p-AMPK/AMPK. This demonstrates that one of the mechanisms VA exerts its antidiabetic properties is via the activation of AMPK [43].

### 4.6. Future Directions

Several studies have proven the antidiabetic properties of *Vernonia amygdalina* in multiple preclinical studies. Its ethnopharmacological use as antidiabetic agent in humans is lacking and, thus, yet to be explored. We suggest that well-designed clinical studies be conducted to validate the antidiabetic properties of VA in human participants in regions where this medicinal plant is frequently used among indigenous communities, such as Ghana and Nigeria. Moreover, the synergistic effect of VA and other known antidiabetic medicinal plants such as *Allium sativum* [86] could be further exploited, as synergistic effect of bioactive compounds from medicinal plants has been proven to be promising and effective [87].

Furthermore, very few of the studies used the identified phytochemicals to confirm the antidiabetic potential of VA. Hence, characterization of the bioactive agents responsible for the antidiabetic activities is warranted. More relevant to the field of drug design, the mechanism of action of these bioactive agents and their synergistic properties could potentially provide a platform for the design of potent antidiabetic drugs from VA in the near future.

Lastly, there is a general lack of data on interaction between VA and approved antidiabetic drugs. Some diabetic patients combine pharmaceutical drugs with herbal medicines such as VA when treating their ailment [88]. However, several herbal medicines, when taken together with Food and Drug Administration- (FDA-) approved antidiabetic agents, could potentially alter their pharmacodynamic and/or pharmacokinetic properties. These interactions can be a double-edged sword, presenting as either improvement in patient outcome or adverse effects (toxic effects) [88]. As such, more comprehensive and rigorous studies are urgently needed to guide clinical practice as well as safeguarding the well-being of diabetes patients.

## 5. Conclusion

Various medicinal plants have been reported to exhibit antidiabetic properties, and VA is no exception. In this review, we summarized the various therapeutic potential of VA in the treatment of diabetes and its associated complications such as nephropathy and dyslipidemia. VA has demonstrated hypoglycemic abilities via several mechanisms in animal models and could therefore be potent in human studies.

## Figures and Tables

**Table 1:** Other names of *Vernonia amygdalina*.

Country	Vernacular names
Common name	Bitter leaf; toothbrush tree
Ghana	Awonwono
Nigeria	Onugbu
Cameroon	Muop; ndole
Uganda	Mululuza
Tanzania	Tuntwano
Rwanda	Umubilizi
Eritrea	Grava
Malaysia	Pokok bismillah

**Table 2:** Scientific classification.

Kingdom	Plantae
Phylum	Spermatophyta
Subphylum	Angiospermae
Class	Dicotyledoneae
Order	Asterales
Family	Asteraceae
Genus	*Vernonia*
Species	*Vernonia amygdalina*

**Table 3:** Studies on the therapeutic role of *Vernonia amygdalina* in diabetes and associated complications.

S/N	Reference	Parts utilized	Solvent for extraction	Dose	Bioactive compound	Experimental model/human	Treatment effect
1	Djeujo et al. [36]	Roots and leaves	Distilled water, ethanol, and Soxhlet	10–100 *μ*M	Luteolin and vernodalol	*In vitro*	*α*-Glucosidase activity, antioxidation, advanced glycation end products (AGEs)
2	Patathananone et al. [37]	Leaves	Methanol, ethanol and acetone, isopropanol, ethyl acetate and hexane	10–100 *μ*l	Flavonoids, phenols, coumarins, saponins, tannins, terpenoids, steroids, and cardiac glycosides	*In vitro*	Antioxidant activity, antityrosinase activity, and antiamylase activity
3	Medjiofack Djeujo et al. [38]	Roots and leaves	Distilled water, ethanol, Soxhlet	5-500 *μ*g/ml	Catechin, caffeic acid, chlorogenic acid, luteolin, vernodalol	*In vitro*	*α*-Glucosidase activity, AGE formation, and oxygen radical absorbance capacity
4	Erukainure and Islam [39]	Leaves	Ethanol, distilled water, ethyl acetate	500 mg/kg	Not reported	Sprague Dawley rats	Antioxidative and antidiabetic
5	Okoduwa et al. [40]	Fresh leaves	Methanol	10 mg/kg b.w	11*β*,13-Dihydrovernolide	Albino Wistar rats	Hypoglycemic effect
6	Erukainure et al. [41]	Leaves	Distilled water	30, 60, 120, and 240 *μ*g/ml	L-Serine, L-cysteine, L-proline, Cumidine, nicotinic acid, isoquinoline, 3-methyl-salicylic acid, and *γ*-octalactone	Sprague Dawley rats	Glucose uptake and neuroprotection
7	Erukainure et al. [42]	Leaves	Distilled water	15, 30, 60, 120, and 240 *μ*g/ml	Acidic lipid, mucilage and pectin, lipids, polyphenols, and alkaloids	Sprague Dawley rats	*α*-Glucosidase activity, DPPH, FRAP, DNA fragmentation, glucose uptake, and absorption
8	Wu et al. [43]	Leaves	Ethanol	50, 100, and 150 mg/kg	Not reported	Kunming mice	Hypoglycemic effect, improved insulin resistance, suppression of gluconeogenesis
9	Aba and Udechukwu [44]	Leaves	Distilled water	200 mg/kg	Saponins, flavonoids, tannins, glycosides, alkaloids, terpenes	Albino Wistar rats	Hypoglycemic effect
10	Okon and Umoren [45]	Leaves	Distilled water	52 mg/kg	Flavonoids, terpenes, polyphenols, alkaloids, saponin, steroids, and cardiac glycosides	Albino Wistar rats	Hypoglycemic effect
11	Okoduwa et al. [46]	Leaves	Methanol	250 mg/kg b.w	Alkaloids, carbohydrates, glycosides, flavonoids, polyphenols, saponins. Steroids, tannins, and tritapene	Albino Wistar rats	Hypoglycemic, antilipidemic, pancreatic *β*-cell regeneration, restoration of biomarkers of liver and kidney function
12	Asante et al. [13]	Young and old leaves	Ethanol	10, 30, and 300 mg/kg b.w	Flavonoids, saponins, alkaloids, saponins, tannins, glycosides, terpenoids, anthraquinones, and reducing sugars	Sprague Dawley rats	Hypoglycemic, antilipidemic, and tissue regeneration
13	Okoduwa et al. [47]	Leaves	Methanol	250 mg/kg b.w.	Carbohydrates, glycosides, cardiac glycosides, saponins, steroids, triterpenes, tannins, flavonoids, alkaloid	Albino Wistar rats	Hypoglycemic effect
14	Atangwho et al. [48]	Leaves	Petroleum ether and chloroform	200 and 400 mg/kg	Not reported	Sprague Dawley rats	Suppression of gluconeogenesis
15	Atangwho et al. [49]	Leaves	Water, methanol, chloroform, petroleum	400 mg/kg	Fatty acids, vitamin E	Sprague Dawley rats	Hypoglycemic effect
16	Akinola et al. [50]	Leaves	Ethanol	400 mg/kg	Polyphenols	Albino Wistar rats	Antihyperglycemic effect
17	Atangwho et al. [51]	Leaves	Ethanol	400 mg/kg b.w.	Alkaloids, glycosides, tannins, saponins, flavonoids, reducing sugars, polyphenols, steroids, hydroxymethyl anthraquinones	Albino Wistar rats	Antihyperglycemic, pancreatic *β*-cell regeneration
18	Akah et al. [52]	Leaves	Methanol	80, 160, and 320 mg/kg	Flavonoids, saponins, carbohydrates, combined and free reducing sugars, tannins, sterols, and balsams	Albino Wistar rats	Hypoglycemic, hypolipidemic
19	Nwanjo [53]	Leaves	Distilled water	200 mg/kg	Alkaloids, carbohydrates, tannins, saponins, flavonoids, and glycosides	Albino Wistar rats	Hypolipidemic and antioxidant
20	Gyang et al. [54]	Leaves	Chloroform	750 mg/kg	Not reported	Albino Wistar rats	Hypoglycemic effect

## Data Availability

All articles used in this review are listed in the reference section of this paper.
